# LVAD as a Bridge to Transplantation—Current Status and Future Perspectives

**DOI:** 10.31083/j.rcm2505176

**Published:** 2024-05-17

**Authors:** Maximilian J. Roesel, Gaik Nersesian, Sebastian Neuber, Henriette Thau, Rosalie Wolff von Gudenberg, Pia Lanmueller, Felix Hennig, Volkmar Falk, Evgenij Potapov, Christoph Knosalla, Jasper Iske

**Affiliations:** ^1^Department of Cardiothoracic and Vascular Surgery, Deutsches Herzzentrum der Charité (DHZC), 13353 Berlin, Germany; ^2^Charité-Universitätsmedizin Berlin, Corporate Member of Freie Universität Berlin and Humboldt-Universität zu Berlin, 10117 Berlin, Germany; ^3^German Center for Cardiovascular Research (DZHK), Partner Site Berlin, 10785 Berlin, Germany; ^4^Department of Cardio-, Thoracic-, Transplantation-, and Vascular Surgery, Medizinische Hochschule Hannover, 30625 Hannover, Germany; ^5^Berlin Institutes of Health at Charité - Universitätsmedizin Berlin, 10178 Berlin, Germany

**Keywords:** heart failure, bridge to transplantation, left ventricular assist devices

## Abstract

Heart failure (HF) is a common disease associated with high morbidity and mortality rates despite advanced pharmacological therapies. Heart transplantation remains the gold standard therapy for end-stage heart failure; however, its application is curtailed by the persistent shortage of donor organs. Over the past two decades, mechanical circulatory support, notably Left Ventricular Assist Devices (LVADs), have been established as an option for patients waiting for a donor organ. This comprehensive review focuses on elucidating the benefits and barriers associated with this application. We provide an overview of landmark clinical trials that have evaluated the use of LVADs as a bridge to transplantation therapy, with a particular focus on post-transplant outcomes. We discuss the benefits of stabilizing patients with these systems, weighing associated complications and limitations. Further technical advancements and research on optimal implantation timing are critical to ultimately improve outcomes and securing quality of life. In a world where the availability of donor organs remains constrained, LVADs are an increasingly important piece of patient care, bridging the critical gap to transplantation in advanced heart failure management.

## 1. Introduction

While the incidence of heart failure (HF) is stable and even appears to be decreasing in developed countries, its prevalence is increasing due to aging, resulting in a significant social and economic burden [1]. Advanced HF is no exception and is becoming more prevalent in both men and women, especially in older age groups, due to improved HF treatment and survival rates [2]. Once pharmaceutical treatments are not sufficient anymore, these patients must rely on either short- or long-term mechanical circulatory support (MCS).

Temporary MCS is used for a few days to several weeks, as a bridge to recovery therapy to support homeostasis until a definitive treatment approach can be applied or a palliative situation needs to be initiated. If short-term MCS does not result in cardiac recovery or clinical improvement, long-term MCS with left ventricular assist devices (LVADs) may be indicated. Originally developed in the context of transplantation as either a bridge to decision, candidacy, or transplantation, the application was rapidly expanded to lifelong support as a destination therapy (DT) to meet the increasing demand for end-stage HF therapy [3]. Durable LVAD remains second to heart transplantation (HTx) as a therapy for end-stage HF [4], and thus, bridge to transplantation (BTT) remains critical for patients on the waiting list, with evidence of significant improvement in mortality and morbidity in this setting [5]. In this review, we discuss the current status and future potential of LVAD implantation in BTT patients.

## 2. The Concept of Bridge to Transplantation LVAD Therapy

The history of MCS traces back to Gibbon’s heart-lung machine invention in the 1950s, enabling intracardiac surgeries. However, the need for prolonged support after cardiopulmonary bypass (CPB) for patients with failed weaning from CPB, led to the development of the first successful LVAD implantation in 1966 for a patient with postcardiothomy cardiogenic shock [6]. Severe heart failure not only affects patients following cardiac surgery but also those ineligible for surgical treatment options who required transplantation. Thus, the concept of BTT was developed to temporarily support heart insufficient patients with ventricular assist devices until they became eligible for HTx or received a suitable donor organ. However, the increasing gap between organ supply and demand, leading to longer waiting times, particularly in Europe, has led to a change in the established dichotomy of BTT and DT [7]. Today, LVAD therapy is often the only therapeutic option for patients already on the waiting list but, who have little chance of receiving a donor organ in time.

According to the 2021 guidelines of the European Society of Cardiology, LVAD should be considered in patients with persistent severe symptoms despite optimal medical and device therapy and a stable psychosocial background and at least one of the following criteria: (i) severely impaired cardiopulmonary performance, (ii) ≥3 HF hospitalizations in the previous 12 months, (iii) dependence on inotropic therapy or temporary MCS, or (iv) progressive end-organ dysfunction. In addition to LVADs, biventricular assist devices and total artificial hearts can also be used as BTT, but this review focuses on LVADs [8]. Similarly, according to the 2022 AHA/ACC/HFSA guideline, durable LVADs should be considered in selected patients with New York Heart Association NYHA Functional Classification (NHYA) class IV symptoms who are considered dependent on intravenous inotropes or temporary MCS [9].

**Fig. 1.**
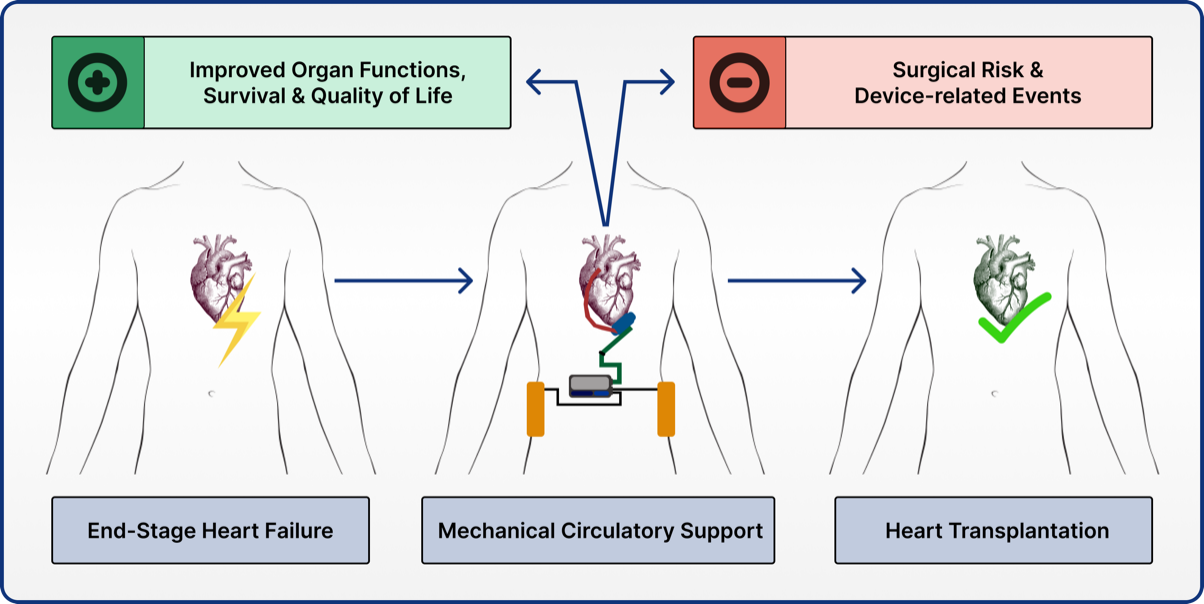
**Mechanical circulatory support as a bridge to transplantation**.

According to the most recent INTERMACS report, survival rates for BTT and BTC patients were 86.5% and 84.3%, respectively [10]. Notably, a significant improvement in the overall survival of LVAD patients has been observed since the implementation of the latest generation of fully magnetically levitated platforms, despite a slight decrease in HTx rates of LVAD patients in recent years [10]. The widespread use of LVAD implantation as a BTT strategy has not only improved the survival rates of patients on the transplant waiting list, but has also enabled candidates to survive long waiting periods [11]. This phenomenon can be explained by the improvement in heart failure symptoms and clinical condition of BTT patients on LVAD support [11]. Despite the increased risk of adverse events (AEs) associated with LVAD support, BTT remains an effective strategy because it reduces the likelihood of death and removal from the waiting list due to clinical deterioration, which is often the only alternative to HTx due to organ shortage [11] (Fig. 1).

## 3. Results of Bridge to Transplant LVAD Therapy

In 1966, the first successful LVAD implantation was performed using a pulsatile extracorporeal system designed by Liotta and DeBakey to provide circulatory support to a patient in cardiogenic shock after cardiac surgery [6]. In the following decades, several devices, including the first total artificial hearts, were tested. However, the potential of these devices for MCS remained limited due to their bulky design and high incidence of complications [12]. In the 1990s, the application of the first successful long-term intracorporeal pulsatile assist devices dramatically changed the field of MCS therapy. Unlike previous models, these devices did not require large control consoles and constant monitoring, allowing patients to be mobile and even discharged with the device [13].

Numerous studies have been conducted to evaluate the safety and outcomes of BTT therapy. The most important results and the limitations of research in this area are presented below (Table 1, Ref. [14, 15, 16, 17, 18, 19]). While there are earlier studies evaluating LVAD as a destination therapy, we are focusing on the studies evaluating it as a BTT [20].

**Table 1.** **Key trials of left ventricular assist devices**.

Author	Device	Study type	n	Findings (BTT)
Miller *et al*., 2007 [14]	HeartMate II	Prospective, multicenter uncontrolled	133	75% of patients were alive at 6 months. 42.1% underwent HTx, 32.3% were still eligible for HTx with ongoing MCS
Pagani *et al*., 2009 [15]	HeartMate II	Prospective, multicenter uncontrolled	281	55.8% of patients underwent HTx within 18 months with a post-transplant survival of 96% (30 d) and 86% (1 yr)
Strueber *et al*., 2011 [16]	HeartWare	Prospective, multicenter uncontrolled	50	Survival at 6, 12, and 24 months was 90%, 84%, and 79%, respectively. Nine patients died (median duration of 94 days)
Aaronson *et al*., 2012 [17]	HeartWare vs. Axial Flow	Prospective, multicenter contemporaneous control	137	Survival at 6 months or explantation in 90.7% of the HVAD group compared to 90.1% in the historical INTERMACS control
Author	Device	Study type	n	Findings (BTT & DT)
Netuka *et al*., 2015 [18]	HeartMate III	Prospective, multicenter uncontrolled	50	BTT (54%) + DT (46%). At 6 months, 88% of patients continued on support, 4% received transplants, and 8% died
Mehra *et al*., 2018 [19]	HeartMate III vs. HeartMate II	Prospective, multicenter randomized control	366	BTT + DT. Overall rate of stroke was lower in HeartMate III group than in axial-flow pump group (10.1% vs. 19.2%)

HeartMate II (Thoratec, St. Jude Medical, Abbott Laboratories). HeartMate III (St. Jude Medical, Abbott Laboratories). BTT, Bridge to transplant; DT, destination therapy; MCS, mechanical circulatory support; HTx, heart transplantation; n, number of study participants; d, day; yr, year; HVAD, HeartWare Ventricular Assist System.

### 3.1 HeartMate II (HM2)

In a first uncontrolled, prospective, multicenter study, 133 patients with end-stage HF on the waiting list for HTx underwent implantation of the HM2, an axial continuous-flow pump [14]. Within 180 days after implantation, 56 (42.1%) underwent heart transplantation, 32 (23.3%) were still on the active transplant list, while 11 (8.3%) were still eligible for HTx including 4 who preferred to continue MCS. Importantly, renal and hepatic function improved during MSC from baseline to 3 months. This was also evident in a functional assessment measured by improved NYHA functional class and 6-minute walk test. Quality of life (QoL) also improved significantly.

In an extension to this trial between March 2005 and April 2008, 281 patients were urgently listed for HTx and underwent implantation of the same device [15]. At 18 months after implantation, 157 (55.8%) patients had undergone HTx with a post-transplant survival rate of 96% at 30 days and 86% at 1 year. Similar to the 3-month results in the first study, both QoL and functional assessment improved significantly between baseline and 6 months. Liver and kidney function also improved significantly.

### 3.2 HeartWare

The third study comprised the initial European evaluation of the HeartWare Ventricular Assist System (HVAD). This device is also a continuous-flow pump, but unlike the HM2, it has a centrifugal flow configuration [16]. The HVAD pump was implanted into 50 heart transplant candidates (NYHA IV). Survival at 6, 12, and 24 months was 90%, 84%, and 79%, respectively. Within 2 years, 20 (40%) patients underwent HTx, 4 (8%) patients had the pump explanted after myocardial recovery, and 17 (34%) patients were still on MCS, while 9 patients (18%) died due to sepsis (3), multiple organ failure (3), and hemorrhagic stroke (3). While bleeding was the most common complication, occurring in 10 (20%) patients within the first 30 days after implantation, it was significantly less common than in previous studies. The small size of the device allows for placement in the pericardial space, reducing the potential for surgical bleeding and device-related infection in the abdominal compartment. In terms of functional assessments, there were no statistically significant declines in neurocognition for any of the cognitive domains from baseline to 1, 3, and 6 months after the implantation. Instead, there were improvements in several cognitive domains.

The first US trial compared 140 patients implanted with the HVAD with contemporaneous control patients derived from the national INTERMACS patient registry who almost received an axial design device such as the HM2 [17]. The success of the HVAD device was found to be non-inferior to that of the controls in both the per-protocol and safety populations. The primary outcome of survival on the originally implanted device, transplantation or explantation for ventricular recovery at 180 days was achieved in 90.7% of the HVAD group compared to 90.1% of the INTERMACS historical control patients. In the HAVD group, 88 (62.9%) patients were still on the originally implanted study device at 180 days with 73 (52.1%) still on the waiting list. 39 (27%) were transplanted during this period [16].

### 3.3 HeartMate 3 (HM3) 

In a single-arm, CE-Mark trial, 50 patients received the HM3 with an indication for BTT (54%) or DT (46%) [18]. The 6-month survival rate was 92%, with 88% of patients continuing LVAD support, 4% undergoing transplantation, and 8% deceased. All patients were in NYHA class IIIB or IV prior to implantation, but improved steadily and significantly at 1, 3, and 6 months. At 6 months, over 80% of patients were in NYHA class I and II. In addition, patients improved significantly on the 6-minute walk test at both 3 and 6 months.

TH Momentum 3 TRAIL which included patients with advanced HF as either BTT or DT, compared 190 patients implanted with the HM3, a centrifugal-flow circulatory pump, to 176 patients implanted with the HM2, an axial-flow pump [19]. The primary endpoint was 2-year survival free of stroke with modified Rankin score of more than 3 or reoperation to replace or remove a malfunctioning pump. 79.5% of the centrifugal-flow pump group versus 60.2% of the axial-flow pump group achieved the 2-year goal, with a significant hazard ratio of 0.46 (95% CI, 0.31 to 0.69) for superiority. Overall, the difference between the groups was driven by the failure to meet the no reoperation endpoint. The rates of death and disabling stroke were similar, but the overall stroke rate was lower in the centrifugal-flow pump group (10.1% vs. 19.2%). In addition, pump thrombosis was suspected in only 1.1% of patients in the centrifugal-flow group versus 15.7% in the axial-flow group. Notably, there was no difference in the achievement of the primary endpoint between the BTT and DT groups. All patients improved on the 6-minute walk test and NYHA functional class, although there was no significant difference between groups. Scores on the KCCQ, EQ-5D-5L, and EQ-5D VAS scores also improved in all groups.

## 4. BTT or Primary HTx

The decisive question is whether it is beneficial to implant an LVAD prior to HTx in terms of pre-transplantation, the procedure itself, and post-transplant outcomes. Today’s devices are more biocompatible, smaller, and more reliable, allowing for greater patient mobility [21]. In addition, as described above, LVAD devices facilitate adequate perfusion and homeostasis, leading to improved organ function, cardiopulmonary performance, and QoL. However, some studies suggest negative effects of long-term use of LVADs on organ function, which is particularly relevant today [22, 23, 24, 25]. To better understand the impact of LVAD use on QoL, hemodynamically stable BTT patients (≥INTERMACS 4) were compared to hemodynamically stable HTx listed patients who were theoretically eligible for LVAD implantation. A total of 21 patients underwent HTx after LVAD implantation (HM2: 2; HM3: 7; Medtronic HVAD: 12). 17 propensity score-matched pairs were created to analyze primarily days alive and out of hospital (DAOH) and secondarily survival at 1-year post-decision. Overall, median DAOH was 281 in the LVAD group and 329 in the HTx group, while the 1- and 3-year survival was 82.4% and 76.5% in the LVAD group, and 76.5% and 58.8% in the HTx group. However, it should be noted that the difference in DAOH was not statistically significant. The median time to death was 401 days in the LVAD group versus 314 days, while the median time to HTx was 256 days and 179 days in the LVAD and HTx groups respectively [26].

Looking at the complexity of the procedure itself, it was found that despite longer cardiopulmonary bypass time, LVAD as a BTT did not adversely affect allograft function, hospital length of stay, or long-term outcomes after HTx [27]. However, according to a more recent multicenter study, prior sternotomy is a risk factor for worse survival after HTx, mainly due to increased early postoperative mortality. However, this is mainly true for patients with previous transplantation and not for LVAD patients. Importantly, a subgroup analysis comparing propensity-matched samples of patients who underwent primary HTx with BTT patients showed no difference in long-term survival. The authors argue that the protective effects of LVAD therapy may counteract the increased operative complexity associated with prior surgery [28]. A recent multivariable analysis even showed that that prior MCS (LVAD and biventricular VAD) was associated with reduced 1-year mortality and comparable 5-year survival rates (65% vs. 60%, respectively) [29]. Another study showed that the BTT is associated with a potentially higher risk of post-transplant mortality, especially within 1 year after transplantation [30]. There are several studies available with different results regarding the postoperative outcomes of BTT patients compared to primary HTx patients [30, 31, 32, 33, 34, 35, 36, 37]. A recent meta-analysis found no difference in outcomes within 5 years between BTT and primary HTx patients [38]. The exact use of LVAD prior to HTx remains to be clarified, especially in the current situation of severe organ shortage and continuous innovations of available devices.

## 5. Complications Associated with Bridge to Transplant LVAD Therapy

Although LVAD implantation appears to offer several therapeutic benefits, it is associated with potential complications. Long waiting times often lead to the occurrence of serious device-related complications (DRC) before an organ becomes available [39]. Careful clinical evaluation is required, but especially in Europe, the complications must often be accepted in order to pave the long road to transplantation. Major AEs include bleeding, device thrombosis, stroke, infection, right HF, aortic regurgitation, ventricular arrhythmias (VA), and psychological distress [40, 41].

Bleeding occurs in 30–60% of the patients after LVAD implantation, in rare cases today at pump connections but most commonly manifesting at mucosal surfaces of the gastrointestinal tract or as intracranial hemorrhage in the brain [41, 42, 43]. In fact, 15–30% of LVAD patients experience gastrointestinal bleeding [44, 45] due to mucosal damage, platelet dysfunction, antithrombotic therapy, and angiodysplasia [46], making it the leading cause of readmission within 30 days of discharge [47]. Interestingly, peri transplant bleeding events were observed to be more frequent in patients with LVAD in T-status than in patients with LVAD in HU-status because of complications related to the device [39], suggesting an acquired von Willebrand syndrome, which is commonly observed in patients with long-standing MCS [48].

While device thrombosis may only affect up to 10% of LVAD recipients within 3 months [49], it can lead to complete pump failure, requiring emergency treatment [50, 51].

In addition, intracranial hemorrhage and stroke are among the leading causes of mortality with LVADs [52, 53, 54]. A study of more than 18,000 LVADs showed that the rate of stroke 1 year after implantation is 13% for axial-flow LVADs and 20% for centrifugal-flow LVADs [53].

Despite all the necessary measures and evaluation of the right heart prior to LVAD implantation, right ventricular failure (RVF) remains a serious complication after LVAD implantation, with rates ranging from 5% up to 44% [43, 44, 45]. Notably, RVF contributes significantly to postprocedural morbidity and mortality as well as eligibility for transplantation due to RVF failure derived end-organ consequences [55]. As the ventricular interaction is altered with LVAD implantation, the combination of multiple mechanisms such as an increased right ventricular (RV) afterload, decreased RV preload or impairment of contractility leads to RVF [56, 57]. The increase in cardiac output from the LVAD results in increased venous return to the RV, possibly exacerbating pre-existing RVF [58, 59]. In addition, excessive displacement of the interventricular septum to the left, especially in the setting of aggressive LV decompression with continuous-flow LVADs, may further reduce the contribution of the septum to RV contraction, leading to RVF [59, 60, 61]. The HeartMate II LVAD study investigating 484 enrolled patients for the occurrence of RVF found that 6% of patients required a right ventricular assist device after LVAD implantation, and 7% of LVAD recipients required extended inotropes and late inotropes, respectively [62]. The occurrence of RVF was associated with worse overall clinical outcomes [62]. Interestingly, women displayed a significantly higher rate of right HF requiring right ventricular assist device (RVAD) implantation [63]. Since 4–6% of patients presenting with RVF after LVAD implantation do not respond to inotropic medical therapy and flow adaptation of the LVAD [57, 59], temporary right ventricular support may be necessary [64]. A study investigating the benefit of early and liberal RVAD treatment in patients who had undergone LVAD implantation and exhibited with RVF risk factors demonstrated comparable clinical outcomes despite severely sicker patients in the RVAD group such as extracorporeal life support or preoperative hemofiltration [65]. In addition, patients who received a temporary RVAD at the same time as LVAD implantation displayed a higher 30-day survival rate compared with patients who received delayed RVAD support [66].

As a further complication following LVAD implantation, approximately 25% of LVAD patients develop aortic regurgitation within the first year after implantation [46, 47, 48, 49], caused by a complex mechanism involving variations in aortic root blood flow and pressure.

The incidence of VA after LVAD implantation ranges from 20% to 60% [67, 68, 69]. Although LVAD patients can tolerate VA, it can contribute to right heart dysfunction, suction events, thrombus formation, and poor perfusion, ultimately leading to impaired blood flow [70, 71, 72]. In addition, LVAD patients may experience psychological distress after implantation, which can affect the patient’s overall health and BTT strategy [73].

Related to the extracorporeal energy delivery, INTERMACS reported that within 1 year of LVAD implantation, the most common infectious complications are pneumonia (23%), sepsis (20%), and driveline site infections (19%), often caused by skin flora [52, 74, 75, 76]. In particular, biofilm formation at the interface of the driveline with the injured skin poses a challenge to eradicating bacterial pathogenesis [77]. According to the European Registry for Patients with Mechanical Circulatory Support, sepsis, along with multiple organ failure, is the leading cause of early mortality in LVAD patients [52, 78, 79]. In the context of prolonged LVAD support, device infection has been found to be the most common LVAD complication leading to high-urgency transplantation [53, 80]. However, the occurrence of LVAD complications does not seem to influence the outcome after HTx [39].

Anti-infective therapeutic protocols may include wound dressing in combination with adjunctive therapies such as vacuum-assisted closure therapy, cold atmospheric plasma, or antibiotic beads [77]. In many cases, however, the therapeutic benefits are negated by recurrent infections associated with biofilm persistence [77]. Alternatively, severe driveline infections can be treated with surgical intervention, such as driveline repositioning followed by wound debridement [77]. Therefore, preventing infection in the first place remains of paramount importance. A number of preventive measures have been incorporated into clinical practice including perioperative antimicrobial prophylaxis and postoperative driveline care management [77]. In a small study, merbromin used for local irrigation showed a significant reduction in the development of infection, with all 31 patients treated being free of infection after LVAD implantation [81]. Other approaches, such as wrapping the silicone driveline with biosynthesized cellulose, have shown a reduction in local bacterial colonization, which, together with the known anti-fibrotic effect of biosynthesized cellulose, may promote more efficient immune clearance after driveline implantation and support the efficacy of local antibiotic treatments [82].

## 6. Future Perspectives and Innovations of BTT

Technical advances have been made in the miniaturization of current devices, allowing for minimally invasive surgical approaches. In particular, the LATERAL trial used a left thoracotomy combined with an upper hemisternotomy or right anterior thoracotomy for minimally invasive LVAD implantation [83, 84]. In addition, preservation of the pericardium around the RV may support right heart function by minimizing RV dilatation and displacement of the heart from the pericardial cavity [85, 86, 87], while allowing primary sternotomy at the time of HTx with reduced adhesions, extensive dissection, and subsequent bleeding [85, 88].

As persistent right heart strain with RVF induced by heart failure with reduced ejection fraction HFrEF is a common complication after LVAD implantation, several risk scores have been developed to predict RVF and improve decision making [89]. For example, a recent study identified the following features to predict RVF: need for vasopressors, aspartate aminotransferase level ≥80 IU/L, bilirubin ≥2.0 mg/dL, and creatinine ≥2.3 mg/dL [89].

As LVAD technologies evolve, novel devices can provide longer support with significantly reduced DRC. For instance, the HM3 centrifugal-flow device with a fully magnetically levitated impeller outperforms its predecessor, the HM2 axial-flow device, particularly in terms of hemocompatibility [90]. The MOMENTUM 3 trial demonstrated this superiority with a 76.9% incidence of stroke-free survival and no reoperations due to pump failure [90]. The HM3’s ability to generate an artificial pulse by sequentially modulating the rotor speed prevents blood stasis and thrombus formation by washout, ensuring longer complication-free support [91]. In fact, patients who received the HM3 without the standard antithrombotic admiration of aspirin experienced less non-surgical bleeding with no increase in the risk of thromboembolism [92].

Other low DRC devices include the EVAHEART®2 left ventricular assist device (EVA2), which was suggested to have the potential of reducing malformations, obstructions, and thrombus formation through the design of its double-cuff, tipless inflow cannula that does not extend into the LV cavity [93]. The potential equivalence of EVA2 to HM3 was most recently evaluated in the COMPETENCE trial with a final report lacking [93].

In addition to technical advances aimed at reducing complications, the timing of LVAD implantation prior to HTx is critical. BTT patients who received HTx within 1 month of implantation showed a significant increase in mortality. Interestingly, there was no significant difference in 30-day, 1-year or 5-year mortality in patients with an LVAD longer than 31 days versus primary HTx [94]. Very early transplanting seems to be unbeneficial too, demonstrated by higher mortality rates in patients transplanted within 7 days of LVAD impanation [95]. On the other end, prolonged LVAD support is also associated with compromised survival compared to shorter LVAD duration after HTx [96]. An analysis showed that patients who had >1 year of LVAD support had reduced 3-year survival after HTx compared to the <1 year support group, possibly due to increased rates of AE [36]. Looking at even longer durations of LVAD support, another study showed that the >2-year LVAD support group had significantly reduced 30-day and 2-year survival compared to the <1-year and 1–2-year LVAD support groups. In addition to the increased risk of AEs, the increased baseline risk may also play a role [97]. Risk factors such as prior valve surgery, prior coronary artery bypass grafting, higher mean arterial pressure, and hypertension seem to influence optimal timing of implantation [98].

Overall, the expected waiting time for HTx plays an additional critical role in decision making with average waiting times of 1 month in the United States versus up to 1 year in Germany. The development of short- and intermediate-term devices is therefore of varying importance in different countries. In the United States, long-term LVADs are rarely used as BTT. Instead, short-term devices such as Micro-axial flow pumps are preferred due to their less invasive nature compared to other MCS devices [99]. For example, Impella 5.5 as a BTT demonstrated a 1-year survival rate of 89.5% [100].

Despite the complexity due to implantation time, technical advances have massively improved the feasibility and outcomes of LVAD patients. In addition, the development of other short-term devices may be beneficial due to the increased mortality in HTx patients who undergo transplantation within 1 month of LVAD implantation.

LVAD therapy is moving towards the use of coplanar energy transfer systems or fully implantable devices [101, 102], both of which would eliminate the driveline of current pumps, thereby reducing the risk of infections. The Arrow LionHeart LVD 2000 (Penn State University, PA, USA) was the first fully implantable system with percutaneous energy transfer technology designed for DT. It demonstrated an 18-month survival rate of 50% in the first six patients, with no system-related problems or device-related infections [103]. Since then, only a few devices, such as the Arrow Lionheart [104] and the Abiocor Total Artificial Heart [105], have achieved clinical relevance. The most recent of these devices to be clinically tested is the Leviticus FiVADTM (Leviticus Cardio Ltd., Petah Tikva, Israel), which uses a novel wireless power transmission system called Coplanar Power Transmission. It consists of two coils: an internal coil located in the lower part of the right pleural cavity and an external coil attached to a power transmission belt. The latter transmits energy by induction to charge the internal battery/controller located in the right lateral chest wall. This system is designed to be compatible with all commercially available LVADs and has been successfully used to date in 2 patients coupled to the Jarvik 2000® LVAD [102]. The performance and durability of fully implantable devices may bring LVAD therapy into the mainstream of clinical practice. This also gives rise to the question if durable LVAD systems may supersede the concept of BTT due to the utilization of ventricular assist devices as DT. Since current studies estimate no significant increase in donor organ supply particularly in Europe, the progressive development of long-term ventricular assist device may cause a reformation of the allocation system in countries with severe donor organ shortness. As the technology improves, LVADs are indeed becoming a viable alternative to HTx in patients with advanced HF [106], with similar 5-year survival rates [107, 108, 109]. If the establishment of long term LVADs should be successful, short-term devices may be of choice for those patients still being elected for HTx as primary therapeutic approach. Indeed, recent studies on the Impella 5.0 and 5.5 for instance, have demonstrated non-inferior performance when compared to durable implanted LVAD systems [110], requiring less invasive implantation techniques while also being associated with excellent survival rates and minimal morbidity post-transplantation [111].

In patients with biventricular heart failure, treatment with an LVAD alone is not always sufficient. Moreover, severe cardiac injury such as a thrombotic aortic root or an infarction derived ventricular rupture may render patients ineligible for LVAD implantation requiring alternative treatment. Therefore, the development of biventricular devices remains of particular importance. Total artificial hearts (TAH), such as the Syncardia TAH, which is currently the only TAH approved by the FDA [112], are being developed to treat such patients. Other clinically tested devices include the Carmat TAH (Carmat SA, Velizy Villacoublay, France), which received BTT approval in Europe in 2020, and was recently successfully implanted prior to HTx [113]. Due to its biologically coated surfaces, it has the potential to eliminate the need for systemic anticoagulation [112]. In addition, development and testing of other TAHs, such as the BiVACOR TAH, is ongoing [114]. In a bovine animal model, it has demonstrated a unique ability to adapt to higher metabolic demands compared to the currently approved MCS devices [112, 115, 116]. Notably, existing TAHs have not yet demonstrated the potential to eliminate the need for HTx, but the question remains whether concurrent biventricular assist device (BiVAD) implantation should be preferred over LVAD in selected high-risk patients [117]. Of note, some patients are not eligible for TAH implantation due to anatomic properties and size-mismatches. The decision for TAH in patients with biventricular heart failure should be carefully considered, but can be based on indications such as RV failure, restrictive cardiomyopathy, hypertrophic cardiomyopathy, any cardiomyopathy with a small (<4.5 cm) LV end-diastolic dimension and contraindications to long-term anticoagulation [118].

## 7. Conclusions

LVAD implantation is an effective bridging strategy to transplantation when long wait times are expected by limiting the side effects of progressive HF. However, early HTx after LVAD implantation is associated with significant side effects, while longer support times are associated with DRC, suggesting that the BTT approach may be particularly beneficial for patients with mid-term wait times. In the near future, short-term MCS devices are expected to be used for longer support periods, potentially replacing LVAD implantation for patients expected to receive an organ offer in the mid-term. Ongoing research focused on device size reduction, improved flow characteristics, wireless power delivery, and TAH technologies may provide an alternative therapy to HTx as the overall shortage of donor organs continues to define the field of transplantation.
